# 1415. Effect of Self-Sanitizing Copper Impregnated Surfaces on Healthcare-Associated Infection Rates

**DOI:** 10.1093/ofid/ofad500.1252

**Published:** 2023-11-27

**Authors:** Piyali Chatterjee, Marjory Williams, Hosoon Choi, John David Coppin, Morgan Bennett, Thanuri Navarathna, Brandon Corona, Angelia Bridges, Munok Hwang, Richard Nelson, Robin Keene, Chetan Jinadatha

**Affiliations:** Central Texas Veterans Health Care System, Temple, Texas; Central Texas Veterans Health Care System, Temple, Texas; Central Texas Veterans Health Care System, Temple, Texas; Central Texas Veterans Health Care System, Temple, Texas; Central Texas Veterans Health Care System, Temple, Texas; Central Texas Veterans Health Care System, Temple, Texas; Central Texas Veterans Health Care System, Temple, Texas; Central Texas Veterans Health Care System, Temple, Texas; Central Texas Veterans Health Care System, Temple, Texas; Salt Lake VA, Salt Lake City, Utah; Central Texas Veterans Health Care System, Temple, Texas; Central Texas Veterans Health Care System, Temple, Texas

## Abstract

**Background:**

Healthcare-associated infections (HAI) are a major cause of morbidity and mortality in the United States. Prevention of HAIs is a multipronged approach that involves hand hygiene, antibiotic stewardship, and environmental sanitation. The efficacy of polymer-based self-sanitizing copper oxide-impregnated solid surface on *Clostridioides difficile (C. diff)* spores in a laboratory setting showed 1-2 log reduction. Here we assessed the efficacy of the same surface for preventing HAIs over a 7-year period in a hospital setting.

**Methods:**

The copper surfaces installation in the hospital began in late 2016 and peaked in January 2018. HAIs that met 2017 National Healthcare Surveillance Network definitions were recorded for all infections between 2014 and 2022. A Bayesian multivariate multilevel negative binomial model was fit to assess the mean HAI rate over time for blood, urine, site specific, pneumonia, and *C. diff* infections, and the effect of copper on those trends while adjusting for autocorrelation, seasonality, average length of stay, and the COVID-19 pandemic. We report the median incidence rate ratio (IRR) and 95% uncertainty interval (UI), as defined by the highest density posterior interval, for the intervention.

Copper Surface Installation

**Figure:**
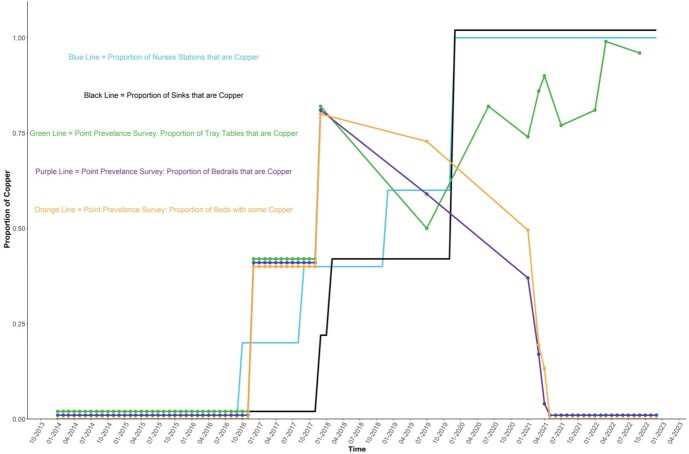

The figure demonstrates the various components of the copper surfaces that were installed in the hospital and the timelines for installation.

**Results:**

During the copper intervention there was 0.36 (0.04 – 0.94) times the incidence for blood, 0.44 (0.06 – 1.26) for pneumonias, 0.61 (0.10 – 1.61) for urine, 0.70 (0.02 – 2.89) for site specific, and 0.85 (0.31 – 1.72) for *C. diff* infections, indicating reduced HAIs during the intervention. Although only the effect for blood infections had a 95% UI excluding an IRR=1, the posterior probability for the model estimates indicates that conditional on this model and data, the chance that the IRR< 1 (indicating reduced HAIs) was 96% for blood, 90% for pneumonia, 80% for urine, 66% for site specific, and 66% for C diff infections.

HAI Rates by time

**Figure:**
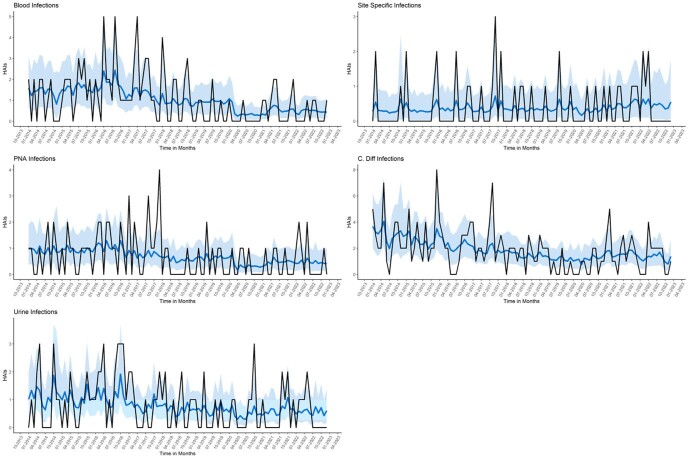

Incident Rate Ratios for HAI and Effect of Copper

**Figure:**
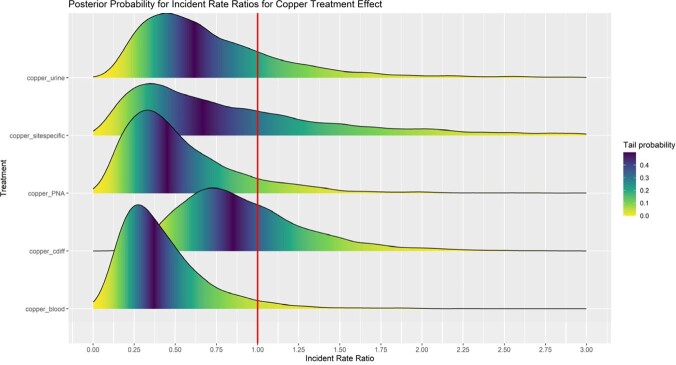

**Conclusion:**

The overall effect of introducing copper bed rails appears to be beneficial with respect to lowering HAIs. The trends were stronger for certain infections such as blood infections and for pneumonias but weaker for *C. diff* infections. Due to this study being a real-world intervention that also overlapped with the COVID-19 pandemic certain beneficial factors may have been overshadowed by the unknowns that the pandemic bought.

**Disclosures:**

**All Authors**: No reported disclosures

